# Peptide nucleic acid (PNA) clamps reduce amplification of host chloroplast and mitochondria rRNA gene sequences and increase detected diversity in 16S rRNA gene profiling analysis of oak-associated microbiota

**DOI:** 10.1186/s40793-025-00674-w

**Published:** 2025-01-28

**Authors:** Usman Hussain, Jim Downie, Amy Ellison, Sandra Denman, James McDonald, Marine C. Cambon

**Affiliations:** 1 School of Natural Sciences, Bangor University, Bangor, UK; 2 School of Biosciences, Institute of Microbiology and Infection, Birmingham Institute of Forest Research, University of Birmingham, Birmingham, UK; 3 Forest Research, Gravel Hill Road, Farnham, UK

**Keywords:** Chloroplast, Mitochondria, Microbiome, 16S rRNA gene sequencing, PCR, qPCR, Bacterial diversity, *Quercus*, PNA clamps

## Abstract

**Background:**

Acquiring representative bacterial 16S rRNA gene community profiles in plant microbiome studies can be challenging due to the excessive co-amplification of host chloroplast and mitochondrial rRNA gene sequences that reduce counts of plant-associated bacterial sequences. Peptide Nucleic Acid (PNA) clamps prevent this by blocking PCR primer binding or binding within the amplified region of non-target DNA to stop the function of DNA polymerase. Here, we applied a universal chloroplast (p)PNA clamp and a newly designed mitochondria (m)PNA clamp to minimise host chloroplast and mitochondria amplification in 16S rRNA gene amplicon profiles of leaf, bark and root tissue of two oak species (*Quercus robur* and *Q. petraea*).

**Results:**

Adding PNA clamps to PCR led to an overall reduction of host chloroplast and mitochondrial 16S rRNA gene sequences of 79%, 46% and 99% in leaf, bark and root tissues, respectively. This resulted in an average increase in bacterial sequencing reads of 72%, 35%, and 17% in leaf, bark, and root tissue, respectively. Moreover, the bacterial diversity in the leaf and bark increased, with the number of ASVs rising by 105 in the leaf samples and 218 in the bark samples, respectively. In root tissues, where host oak chloroplast and mitochondria contamination were low, alpha and beta diversity did not change, suggesting the PNA clamps did not bias the bacterial community.

**Conclusion:**

In conclusion, this study shows that PNA clamps can effectively reduce host chloroplast and mitochondria PCR amplification and improve assessment of the detected bacterial diversity in *Quercus petraea* and *Quercus robur* bacterial 16S rRNA gene sequencing studies.

**Supplementary Information:**

The online version contains supplementary material available at 10.1186/s40793-025-00674-w.

## Background

The plant microbiome embodies a diverse assembly of microorganisms, forming an ecosystem residing on or within plant tissues with many components providing many benefits to plant health. For example some plant-associated microbiomes have exhibited disease suppressive properties against soil-borne fungal pathogens in various tree species [1], others have conferred resistance against bacterial pathogens in leaves [2] or suppressed root rot [3]. Disease suppression can arise from the production of antibacterial and antifungal substances, or through competition for space and resources [1]. Plant associated microbes can also improve tree health through assisting acquisition and transforming nutrients into plant available forms [4, 5], or by interacting with the plant immune system and modulating hormonal signalling [6].

PCR amplification and DNA sequencing of phylogenetic marker genes, such as the 16S rRNA gene of bacteria and Archaea, is a broadly applied method for inferring microbial community composition [7]. However, 16S rRNA gene PCR amplicon studies of the microbiome of plant tissues, such as the foliage and bark, can be challenging due to the co-amplification of eukaryotic host-derived chloroplast and mitochondria rRNA gene sequences [8, 9]. Eukaryotic chloroplasts and mitochondria evolved from engulfed prokaryotes that originally existed as independent organisms, eventually establishing an endosymbiotic relationship [8, 10]. Due to the evolutionary origin of these eukaryotic organelles, they possess the same conserved 16S rRNA region as prokaryotes and thus are amplified during PCR and can be detected in large abundances in amplicon sequencing data [8, 10].

In plant microbiome studies, the average level of host plastid PCR amplicons in roots across 32 plant species was 23%, but certain plant families such as Asteraceae exhibited contamination ranging from 62 to 94% [11]. The abundance and size of chloroplasts per cell are generally greater in photosynthetic regions [12], therefore host plastid PCR amplification is anticipated to be higher in tissues from above-ground compartments. This has been a hurdle in many plant microbiome studies [13, 14] and has resulted in low relative abundances of bacterial 16S rRNA gene sequence reads, in some cases as low as 0.4% for olive bark samples [15]. Such low relative abundances of bacterial sequencing reads, diminishes the cost-effectiveness of sequencing technologies, are often inadequate for downstream processing, and lead to underrepresentation of the bacterial diversity of the study system [16]. Therefore, by preventing or suppressing host DNA amplification the best possible information on bacterial communities in the plant microbiome would be obtained.

Using mismatching primers is one way of suppressing amplification of host DNA. Mismatching PCR primers (e.g. 799 F) contain base pair mismatches with eukaryotic chloroplast sequences [17], but retain specificity for prokaryotic 16S rRNA genes, thereby reducing PCR amplification of chloroplast 16S rRNA gene sequences [9]. Many studies have investigated and compared numerous mismatching primer combinations for their ability to reduce host chloroplast and mitochondria amplification [9, 15, 18]. Cases where these primers were unsuccessful in reducing host 16S rRNA gene PCR amplification include when 341F-783Rabc was tested against high abundances of chloroplast sequences [19]. Additionally, using 799 F with various reverse primers against certain species such *Acer negundo* and *Ulmus pumila* [20] was reported to lead to very minimal amplification of plant-associated bacterial 16S rRNA gene sequences. Mismatching primers may also fail to amplify bacterial targets and underrepresent certain phyla, such as *Verrucomicrobia* and bacteria with photosynthetic abilities such as *Chloroflexi* and *Cyanobacteria* [9, 21, 22]. These amplification biases have been demonstrated to influence bacterial clustering patterns in beta diversity analysis [23], thus potentially compromising the comparability of data across studies. Often a choice must be made between using mismatching primers to exclude host DNA or using universal primers such as 515F-806R which have been shown to uncover greater microbial species diversity and richness [24, 25] but with a risk of obtaining reads from host chloroplast and mitochondria.

Peptide Nucleic Acid (PNA) clamps are nucleic acid analogues in which the normal sugar phosphate backbone found in DNA and RNA is replaced by amide-linked N-(2-amino-ethyl)-glycine units [26]. These synthetic molecules follow the same DNA Watson and Crick binding patterns but possess a heightened affinity for DNA [27]. If used during PCR, PNA clamps can act as inhibitors, preventing the amplification of specific DNA strands [27]. PNA clamps can prevent PCR amplification by either binding to the primer site and blocking primer binding, or binding within the amplified region to stop the function of DNA polymerase [27, 28]. Studies have compared universal primers with PNA clamps against mismatching primers [29] and have found both methods to reduce levels of host plastid PCR amplification. The mismatching primers generally showed greater reduction in host plastid amplification [19], but the use of PNA clamps led to less taxonomic bias and distortion, and still resulted in at least a 20-fold increase in bacterial 16S rRNA gene reads [29]. However, the efficiency of PNA clamps can vary between plant species and tissue type, as the number of chloroplast and mitochondria per host cell and the 16S rRNA gene sequence may differ [30]. As a result, it is important to check the efficiency of PNA clamps on a case-by-case basis.

As the main aim of this study was to obtain the most accurate indication of the composition of bacterial communities in different oak tissue types, we decided that the PNA clamps were the best tool to use. We therefore aimed to develop and adapt PNA clamps to facilitate bacterial 16S rRNA gene amplicon-based analysis of the oak (*Quercus*) microbiome in above- and below-ground tissues. We assessed the effectiveness of a universal plant chloroplast PNA clamp and a new *Quercus sp*. mitochondria clamp designed in this study. These clamps were used to minimise the amplification of host chloroplast and mitochondria DNA in the oak species *Quercus robur* and *Q. petraea*, and their performance was evaluated using 16S rRNA gene amplicon sequencing and probe-based qPCR TaqMan assays.

## Methods

### Sample collection

Oak leaf, bark and root samples were collected from *Q. petraea* and *Q. robur* from 4 sites in the UK. All *Q. petraea* samples were collected from three 42-year-old trees from a woodland plot in Little Snoring, Fakenham (52.869721, 0.915663) in October 2020. Samples of *Q. robur* were collected from three sites across the UK with one tree being sampled from each site in July 2021. Samples were collected using pre-sterilised equipment (described below). New gloves were used between each tree, while the equipment was cleaned with a 10% bleach solution, followed by rinsing with water. All oak tissue samples were immediately frozen in the field using either liquid nitrogen (for *Q. robur samples*) or dry ice (for *Q. petraea* samples). Samples were maintained on dry ice and transferred to a -80 °C freezer within 5 days of collection and remained frozen until sample lysis and DNA extraction.

### Leaf collection

Oak leaf samples were collected by tree climbers from the upper third portion of the tree crown from both the north and south aspects of the tree. Using a handheld 6 mm hole punch (Trixes), three 6 mm discs were collected from each of 5 leaves from the north and south side of the tree. The three discs were collected from the middle of the central vein and non-vein areas equidistantly in a diagonal pattern. Discs were pooled into a sterile 2 mL screw cap tube, resulting in a total of 30 × 6 mm discs from an individual tree (10 x leaves, 5 from the North side, 5 from the South side, 3 discs per leaf = 30 discs) which represented approximately 0.2 g of oak leaf material per tree.

### Bark collection

Bark cores were obtained using a 10 mm Osbourne Arch Punch (OAP) at a height of approximately 1.3 m. The OAP was placed in between bark plates and hammered with a nylon mallet, the OAP was hammered until a pitch change was heard (this is the sapwood layer being reached) and then hammered a further 5–10 mm into the tree. The cylindrical core was removed into a sterile plastic tray, and the phloem and sapwood layers were separated with forceps. One quarter of each of the phloem and sapwood layers were cut with sterile secateurs and pooled into a sterile 2 mL screw cap tube. This was repeated for the north and south side of each tree, resulting in four equal quarters (2 x phloem and 2 x sapwood, one of phloem and sapwood from each aspect) which represented approximately 0.2 g of inner bark material per tree.

### Root collection

Soil cores were obtained using a sterilised Dutch soil auger, reaching a depth of 30 cm, at approximately 1 m from both the north and south sides of each tree trunk. The cores were placed into sterile plastic trays and fine oak roots selected and shaken lightly to remove excess soil. The fine roots with attached soil from the north and south aspects per tree were then combined with equal weight into a 2 mL screw cap tube, resulting in a total weight of 0.5 g of root material per tree.

### Oak tissue homogenisation

Before DNA extraction, the samples were mechanically lysed via bead-beating. Two sterile 3 mm steel ball bearings were added to each tube, with the addition of 500 mg of sterile 425–600 μm acid-washed glass beads (Sigma– G8772) for root samples. The tubes were frozen with liquid nitrogen for 5 s and bead-beaten in a MOBIO™ Powerlyzer 24 at 2.5 m/s for 20 s, followed by a 10 s resting period to minimise heat to the samples, and subsequently bead-beaten for another 20 s. The tubes were centrifuged for 5 s at 5000 rcf to recollect the tissue, and the process was repeated once more. *Q. robur* bark samples were first homogenised with a mortar and pestle using liquid nitrogen prior to bead-beating; and *Q. robur* root samples followed 4 bead-beating cycles instead of two.

### DNA extraction from oak tissue

DNA was extracted from root samples using the protocol described in Griffiths et al., (2000). Briefly, 0.5 mL of 5% CTAB/phosphate (120 mM pH 8.0) extraction buffer and 0.5 mL phenol/chloroform/isoamyl-alcohol (25:24:1) were added to the lysed samples with beads and bead beaten at 2.5 m/s for 30 s. The tube was centrifuged for 5 min at 16 000 rfc, and the aqueous layer was transferred to a new tube where an equal volume of chloroform/isoamyl-alcohol (24:1) (Sigma - C0549) was added. Tubes were vortexed, centrifuged for 5 min at 16 000 rcf and the aqueous layer was removed and transferred to a new tube. Here, 2 volumes of Polyethylene glycol (PEG) solution (30% PEG 6000 / 1.6 M NaCl) and RNAse A (EN0531) at 100 U/mL were added and incubated at room temperature for 2 h to precipitate the nucleic acids. The DNA was pelleted through centrifugation for 10 min at 16 000 rcf, cleaned with 70% ethanol, dried for 20 min at room temperature before eluting in 50 µL of IDTE (10 mM Tris, 0.1 mM EDTA) pH 7.5 (Integrated DNA Technologies, Coralville, Iowa, USA).

Leaf and bark samples were extracted as follow: an extraction buffer (800µL for *Q. petraea* or 1 mL for *Q. robur*) (pH 8.0) containing 4% CTAB, 1% Polyvinylpyrrolidone (PVP), 0.2 M Tris-HCL, 1.4 M NaCl, and 20 mM EDTA was added to the lysed samples and heated at 60 °C for 60 min, vortexed every 15 min. For *Q. petraea* samples the homogenate was cooled to 37 °C and RNase A (EN0531) at 100 U/mL was added and incubated at 37 °C for 20 min for samples; *Q. robur* samples included a 5 min centrifugation step at 15 000 rcf to separate the aqueous layer prior to the addition and incubation of RNAse A. To clean the aqueous layer of organic matter an equal volume of chloroform/isoamyl alcohol (24:1) was added and mixed by inversion, followed by centrifugation for 15 min at 15 000 rcf; the cleaning step was repeated until the aqueous layer was clear. Subsequently, 50 µL of 3 M sodium acetate (Sigma - S2889), 500 µL of 100% isopropanol (Sigma - I9516) and 250 µL of 6 M NaCl were added and left to incubate at -20 °C for 30 min to precipitate the nucleic acid. The nucleic acid was pelleted for 15 min at 15 000 rcf and cleaned with 70% ethanol, eluted in 50 µL IDTE pH 7.5 and stored at -80 °C for long-term storage.

### PNA clamp design

The universal PNA clamps (Lundberg et al., 2013) for chloroplast (5’– GGCTCAACCCTGGACAG– 3’) and mitochondria (5’– GGCAAGTGTTCTTCGGA– 3’) were aligned with the most abundant chloroplast and mitochondria ASV sequences from preliminary *Q. petraea* 16S rRNA gene sequencing data, shown in table S1, using SeaView 5 muscle alignment v3.8.31. The complement of the universal chloroplast clamp sequence matched 100% with the chloroplast ASV sequence, however the universal mitochondria clamp sequence contained 6 bp mismatches against the mitochondria ASV sequence. A single base pair mismatch can significantly reduce PNA clamp effectiveness [31], so a new PNA clamp, named qmPNA, was designed to specifically target this mitochondria ASV.

Using SeaView 5 muscle alignment the mitochondria ASV was aligned with the 20 most abundant bacterial ASVs from the preliminary dataset, and a 17 bp sequence (5’ - GTGAATTGGTTTCGAGA– 3’) was identified to be present in the mitochondria sequence but absent from the bacterial sequences and thus suitable for the qmPNA clamp design. Two lysine’s was added to the 5’ of the sequence to improve solubility and all PNA clamps were synthesised and ordered from PNA Bio (Newbury Park, USA).

Further in silico analyses were conducted in R (version 4.1.2) to evaluate the specificity of the designed PNA clamps to the chloroplast, mitochondrial, and bacterial ASVs obtained in this study, and preliminary amplicon sequencing data. This was done using the vcountPattern function from the Biostrings package (version 2.62.0) [32]. To generate mPNA clamp sequences for future studies, short DNA sequences (k-mers) were derived from the mitochondrial ASVs. These k-mers were then assessed for their binding specificity, ensuring they bound with 100% specificity to as many mitochondrial sequences from the host as possible while avoiding binding to bacterial sequences. The Biostrings package and base R was used to carry out these evaluations. These mPNA clamps were then tested for their potential of hairpin loop formation using the online PNA Tool from PNA Bio.

### 16 S rRNA gene PCR amplification and sequencing library preparation

The V4 hypervariable region of the 16S rRNA gene was amplified with the universal primers 515F (5’– GTGYCAGCMGCCGCGGTAA − 3’) and 806R (5’- GGACTACNVGGGTWTCTAAT- 3’) [16]; the primers contained a spacer, and tag for demultiplexing, details are shown in table S2. To reduce the impact of PCR inhibitors DNA extracts were diluted (1:50 for leaf and bark, 1:100 for root) in nuclease-free water (Integrated DNA Technologies). To test the effectiveness of the PNA clamps, two PCR treatments were applied for each diluted DNA extract: PCR without PNA clamps, and PCR including both the pPNA and qmPNA clamps. Negative controls with no DNA template were also included for both treatments. PCR was performed in 25 µL reaction volumes using 1x GoTaq^®^ G2 colourless master mix (Promega). The final concentrations were 0.7 µM each of forward and reverse primers, and 1 µM of PNA clamps (if used). Additionally, 2 µL of DNA template was added to each reaction. The PCR cycles included initial denaturation at 94 °C for 3 min, followed by 35 amplification cycles of denaturation at 94 °C for 15 s, PNA clamping at 68 °C for 10 s, primer annealing at 50 °C for 10 s and extension at 68 °C for 20 s, with a final extension at 72 °C for 5 min. PCR amplicons were visualised using 1% agarose gel electrophoresis and quantified using the high-sensitivity dsDNA Qubit kit (Qiagen). PCR products were cleaned with Beckman Coulter™ AMPure XP reagent at 0.9X bead/sample ratio (following the manufacturer’s instructions) and eluted in 20 µL nuclease free water. A maximum of 37 ng of DNA per cleaned PCR sample was pooled with a final concentration of 21.5 ng/µL. The sequence pool was sent for library preparation and Illumina PE250 sequencing on the NovaSeq 6000 SP (Novogene, Oxford).

### Quantitative PCR

qPCR was performed on cleaned PCR products to quantify the absolute DNA copy numbers of host chloroplast and mitochondria after 16S rRNA gene amplification. Chloroplast and mitochondria sequences were quantified using probe-based assays in singleplex reactions using the QuantiNova Probe PCR kit (Qiagen). TaqMan probes were designed with the same sequences as the PNA clamps /56-FAM/GG CTC AAC CCT GGA CAG /3BHQ_2/ and /5HEX/GT GAA TTG GTT TCG AGA /3BHQ_2/ for chloroplast and mitochondria, respectively (Integrated DNA Technologies). The total number of 16 S rRNA gene amplicons (bacteria, chloroplast, mitochondria) was also quantified using 515 F and 806R primers [16] and the QuantiNova SYBR green PCR kit (Qiagen), with three replicate qPCR assays per sample. qPCR reactions were conducted in 10 µL volumes in 384 well plates. Final concentrations of the primers were 0.7 µM and probes were 0.6 µM. The QuantStudio 6 was used and the ROX reference dye and cycling conditions followed the manufacturer’s guidelines. Standard calibration curves for absolute gene quantification were generated from 10^8^ to 10^3^ copies/µL using gBlock™ Gene Fragments (Integrated DNA Technologies); the sequences of the gBlocks included the most abundant bacterial and host chloroplast and mitochondria 16S rRNA gene ASVs from the preliminary data, shown in table S3. No template controls for each assay were included and all assays had three technical replicates. qPCR data was observed and analysed using the QuantStudio 6 and R v4.1.2. Data points where the standard deviation in Ct value for the three technical replicates was greater than 0.4 were removed. Samples for which the SYBR green assay failed were also removed along with their corresponding TaqMan assays. Statistical differences were calculated using the Wilcoxon rank-sum test.

### Bioinformatic analysis of 16S rRNA gene microbial community profiles

A total of 22 260 012 raw fastq sequences were obtained from sequencing. Sequences were demultiplexed using only the tags and Cutadapt [33] with a 0% error rate and no insertions and deletions. After demultiplexing, the dada2 pipeline was followed in R with the dada2 package v1.22.0 [34] to generate amplicon sequence variants (ASVs). The filterAndTrim function was first used to remove reads which contained ambiguous nucleotides. This was followed by Cutadapt to remove the forward and reverse primers from the sequences using default parameters. The filterAndTrim function was used again to remove reads with more than two expected errors, the first instance of a quality of score of less than 2, < 75 bp in length and reads which matched with the PhiX genome. After filtering, ASVs were inferred, the forward and reverse reads were merged, merged reads shorter than 225 bp and longer than 275 bp were removed, chimeras were removed and ASVs were collapsed. Taxonomy was then assigned via RDP Naive Bayesian Classifier algorithm [35], using the SILVA reference database v138 [36]. Subsequently, a phyloseq object was created using the phyloseq package v1.38.0 [37] and ASVs were pruned if none of the samples had over 100 reads for that ASV. The data was then corrected for tag jumping (details shown in supplementary information) leaving 5 848 562 reads and 2722 ASVs for analysis. Summary of read loss through filtering steps is shown in the figure S1. The rarefy_even_depth function from the Phyloseq package addressed differing sequencing read depth. For each sample, the reads with and without clamps were rarefied to the lowest number to allow for comparison. The results from this are shown in Figure S3. The unrarefied dataset was focussed on in this study as the extreme differences in bacterial sequencing read depth led to considerable losses in data after rarefying.

### Statistical analysis and generating plots

The full dataset was first used to illustrate the percentage change of host chloroplast, mitochondria and bacterial 16S rRNA gene reads between samples without PNA clamps and with pPNA + qmPNA clamps added during PCR. Then, reads associated to host chloroplast and mitochondria were removed from the dataset for subsequent analysis. The phyloseq [37] and ggplot2 v3.5.0 [38] packages were used to calculate diversity indices and create graphs. Alpha diversity was estimated using the Shannon index, and sample dissimilarity was estimated using the Bray-Curtis distance after data transformation to ASV relative abundances. Beta-diversity was then visualised using Non-metric Multi-Dimensional Scaling (NMDS) plots. Significant differences in alpha diversity were identified using the Wilcoxon rank-sum test. Dissimilarities in beta-diversity was analysed with a Permutational Multivariate Analysis of Variance (PERMANOVA) test (adonis function from the vegan package v2.6.4 [39]). The tree species, tissue type, and PNA clamp treatment were included as explanatory variables, and 999 permutations were performed.

## Results

### PNA clamps reduce host chloroplast and mitochondria contamination and increase abundance of bacterial 16 S rRNA gene reads

**Fig. 1:**
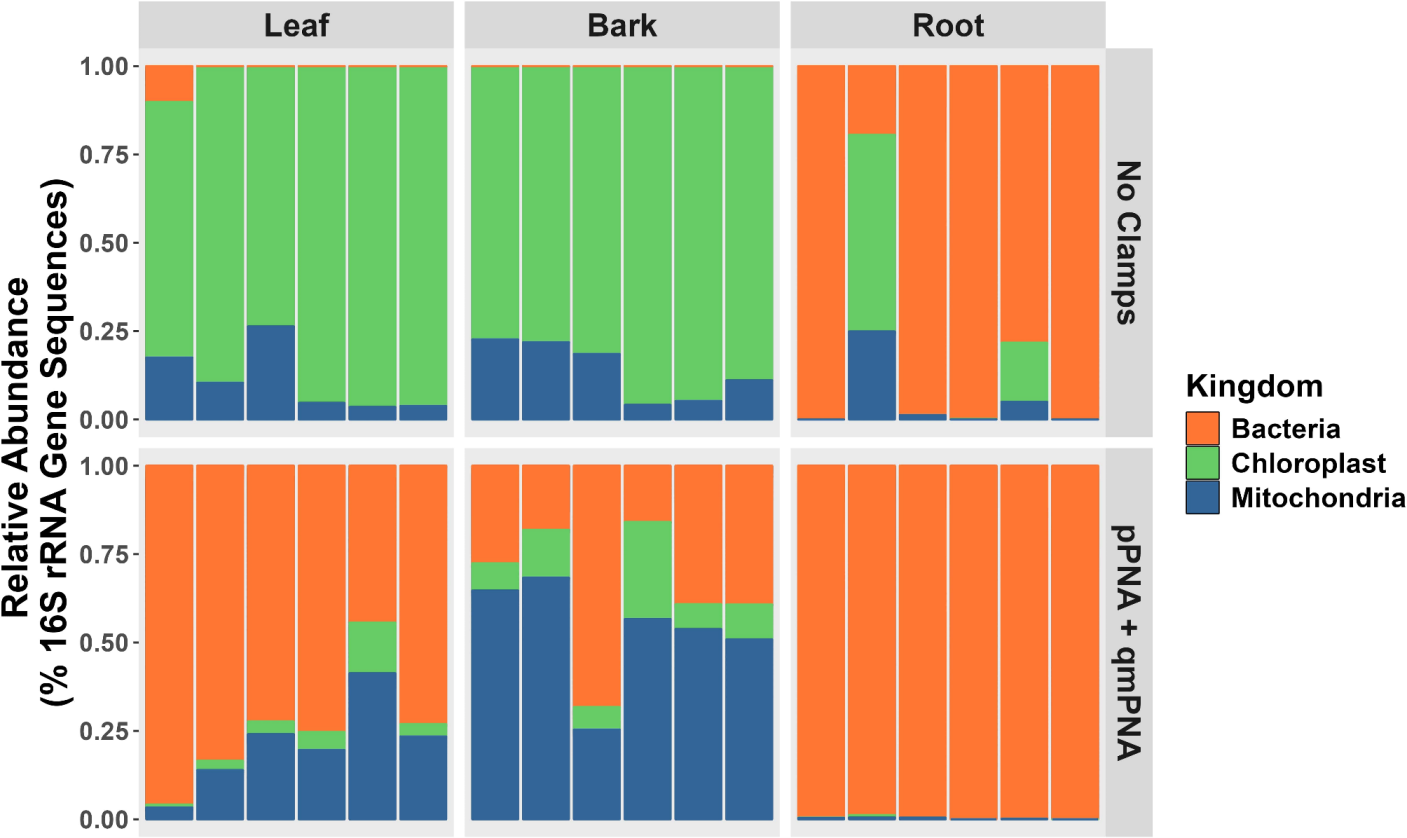
Relative abundance of bacteria, host chloroplast and host mitochondria sequencing reads in oak tissue samples, without and with the inclusion of pPNA + qmPNA clamps during PCR. Comparing the percentage relative abundances of 16S rRNA gene sequences from oak leaf, bark, and root samples under two conditions: samples where no PNA clamps were added during PCR, and samples where both pPNA + qmPNA clamps were included. The relative abundances of amplicon sequence variants (ASVs) identified as host chloroplast (green) and mitochondria (blue) are presented alongside plant-associated bacterial sequences (orange). Each bar represents one tissue sample from one tree. For each tissue type the first three bars represent *Q. petraea* from the Little Snoring site, while the last three bars represent *Q. robur* from three different sites

A total of 5 848 562 filtered 16S rRNA gene reads assigned to 2722 ASVs was obtained after amplicon sequencing, averaging 162 443 (± 94 211) reads per sample with a median of 147 044 reads per sample. The proportion of host chloroplast and mitochondria to bacterial reads, and the effect of PNA clamps varied between tissue types. In samples where PNA clamps were excluded during PCR, reads assigned to host chloroplasts made up a total of 88%, 86% and 11% of all reads in leaf, bark and root samples respectively; and host mitochondria made up 11%, 14% and 5% of all reads in leaf, bark and root samples respectively (Fig. 1). Therefore, the total number of bacterial reads in PCR assays with no PNA clamps added was 1%, < 1% and 84% of all reads within leaf, bark and root samples respectively. In contrast, with PNA clamps included during PCR amplification, host chloroplast made up 4%, 9%, and < 1% of all reads within leaf, bark and root samples respectively; and host mitochondria made up 17%, 45%, < 1% of all reads within leaf, bark and root samples respectively. Therefore, the total number of bacterial reads in PCR assays with the pPNA + qmPNA clamps included was 79%, 45%, and 99% of all reads within leaf, bark and root samples respectively (Fig. 1). When adding only pPNA clamps to the PCR, host mitochondria reads represented 96% (± 3.6%) and 98% (± 3.4%) of reads for leaf and bark, respectively, confirming the reduction of mitochondrial reads by the addition of qmPNA clamps (Figure S2).

Following PNA clamp treatment, there was an average decrease in host chloroplast reads of 82% (± 10%), 74% (± 8%), and 11% (± 22%) in leaf, bark, and root samples, respectively. For mitochondria, there was a reduction in host reads of 5% (± 10%) in root samples and an increase of 10% (± 18%) and + 39% (± 17%) in leaf and bark samples respectively. Additionally, bacterial reads increased by 72% (± 15%), 35% (± 19%), and 17% (± 32%) in leaf, bark, and root samples, respectively.

Of the 2722 unique ASVs identified, 61 were assigned to chloroplasts and 93 to mitochondria. In silico analysis revealed that the pPNA clamp matched with 100% specificity to 10/61 chloroplast ASVs and qmPNA clamp to 18/93 mitochondrial ASVs. In PCR assays where no PNA clamps were added, one host chloroplast ASV accounted for 99% of all chloroplasts reads and one host mitochondria ASV accounted for 97% of all mitochondria reads. The sequences for these most abundant chloroplast and mitochondria ASVs are shown in table S1, and the PNA clamps matched with 100% specificity in silico to these ASVs. Following PNA clamp treatment, these most abundant ASVs accounted for 0% of all chloroplast reads and 62% of all mitochondrial reads. Refer to Figure S2 to observe the impact of omitting the mitochondrial PNA clamp during PCR.

### The addition of PNA clamps during PCR increases bacterial diversity and does not bias microbiome composition

**Fig. 2:**
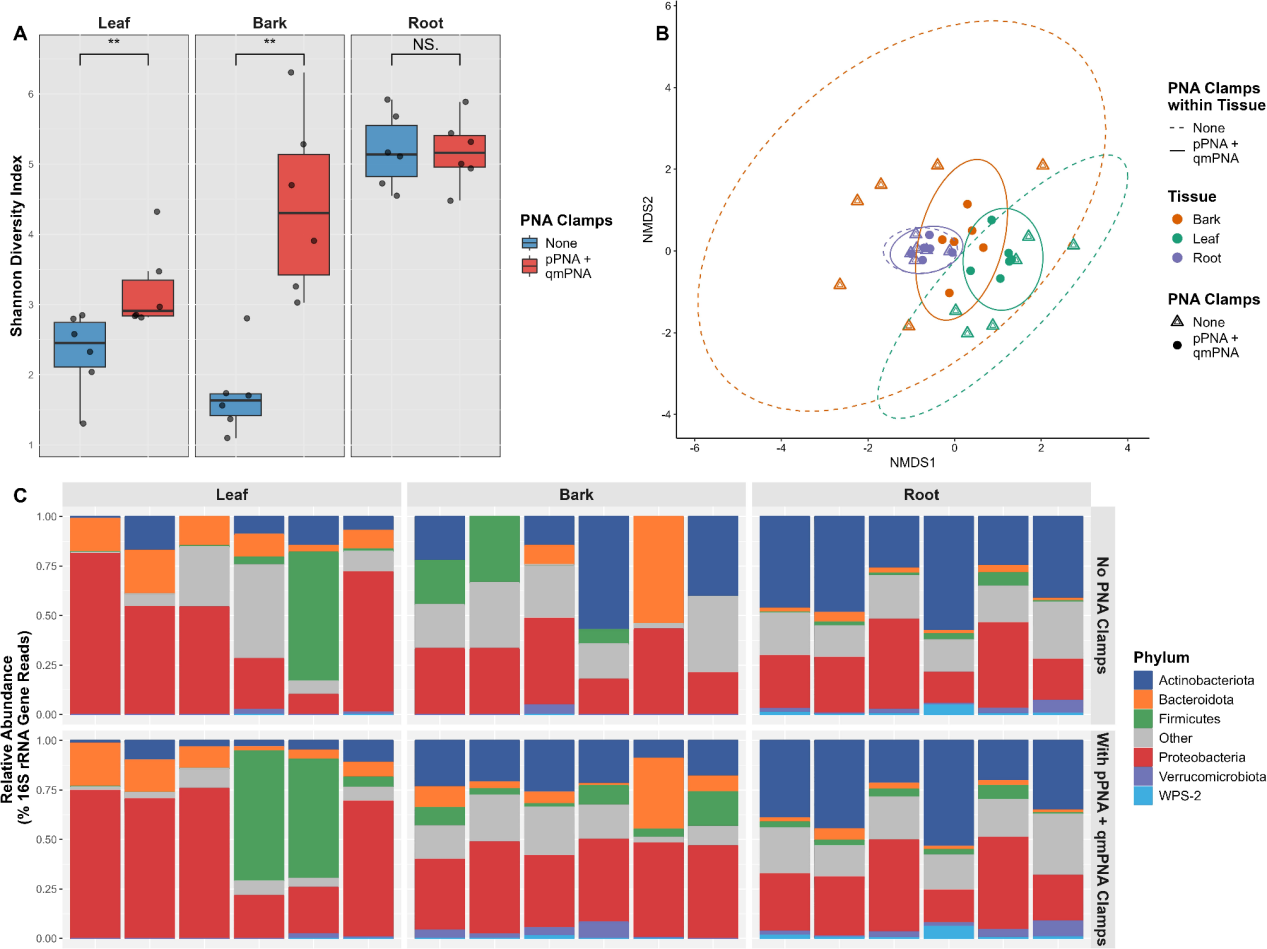
Bacterial diversity and community profiles in oak tissue samples following 16S rRNA gene amplification with and without PNA clamps. All reads assigned to host chloroplast and mitochondria were removed before analysis. **A**. Shannon diversity index. Stars indicate significant differences (Wilcoxon rank sum test. Leaf: p-value = 0.0087; Bark: p-value = 0.0022, Roots; p-value = 0.94) **B**. Bray-Curtis Non-metric multidimensional scaling (NMDS). Solid (no PNA clamps) and dashed (pPNA + qmPNA clamps) ellipses represent 95% confidence intervals of normal multivariate distributions. **C**. Phyla relative abundance based on 16S rRNA gene reads. The top 6 most abundant phyla are presented, and the remaining phyla are categorised into Other (grey). Each bar represents one tissue samples from one tree. For each tissue type the first three bars represent *Q. petraea* from the Little Snoring site, while the last three bars represent *Q. robur* from three different sites

To observe changes in bacterial community compositions between samples when either no PNA clamps or both pPNA + qmPNA clamps were included during PCR, all reads associated with host chloroplasts and mitochondria were filtered out. Alpha and beta diversity analyses were then performed exclusively on bacterial sequences. The Shannon alpha diversity was significantly higher with the addition of pPNA + qmPNA clamps for both leaf and bark samples. (Fig. 2-A). However, there was no significant change in alpha diversity in the root samples (Fig. 2-A). The number of ASVs in samples without the addition of PNA clamp during PCR were on average 21, 8, and 498 for leaf, bark, and root samples, respectively. With the addition of the PNA clamps, the average number of ASVs increased to 126, 226, and 509 for leaf, bark, and root samples, respectively.

Beta diversity analysis revealed that tissue and species significantly influenced the bacterial community composition, while the use of PNA clamps and the interaction between PNA clamps and tissue did not have a significant impact (Fig. 2-B, Table 1).

**Table 1:** **Results of PERMANOVA Analysis on a Bray-Curtis Dissimilarity Matrix generated from Oak Tissue Samples with and without PNA clamps.** The effects of clamps, tissue, species, and their interactions on the Bray-Curtis dissimilarity matrix generated from oak tissue samples following PCR 16S rRNA gene amplification with and without PNA clamps are presented. The columns include the source of variation, degrees of freedom (df), R-squared value (R²), and the p-value for each term in the model. The analysis included 999 permutations

Variable	Df	*R*²	*P*-Value
Clamps	1	0.031	0.12
Tissue	2	0.19	0.001
Species	1	0.050	0.001
Clamps: Tissue	2	0.044	0.633
Residuals	29	0.69	

The total variation explained by the model was 31%, with tissue accounting for most of the variation (19%). To further investigate the effects of clamps and species within the subsets of tissue, separate PERMANOVA analyses were conducted for each tissue type. This revealed for leaves the clamps (R² = 0.097, p-value = 0.14) was not significant but species (R² = 0.23, p-value = 0.001) was significant. Within the bark samples, the clamps (R² = 0.11, p-value = 0.025) and species (R² = 0.11, p-value = 0.027) were significant. In the root samples, the clamps (R² = 0.044, p-value = 0.91) was not significant and the species (R² = 0.18, p-value = 0.047) was significant.

A deeper analysis on the bacterial phyla community composition of oak leaf, bark and root samples with and without PNA clamps during PCR amplification was also conducted and illustrated via a relative abundance graph as shown in Fig. 2-C. In the ‘no PNA clamp’ group, Actinobacteriota (43.25%), Proteobacteria (25.64%), Acidobacteriota (15.05%), Planctomycetota (3.66%), and Verrucomicrobiota (3.24%) were the dominant phyla across all samples (Fig. 2-C). In contrast, the ‘pPNA + qmPNA clamp’ group featured Proteobacteria (45.98%), Actinobacteriota (22.09%), Acidobacteriota (8.64%), Bacteroidota (8.48%), and Firmicutes (4.32%) as the top 5 phyla (Fig. 2-C).

### PNA clamps significantly reduce the absolute abundance of host chloroplast but not host mitochondria sequences

**Fig. 3:**
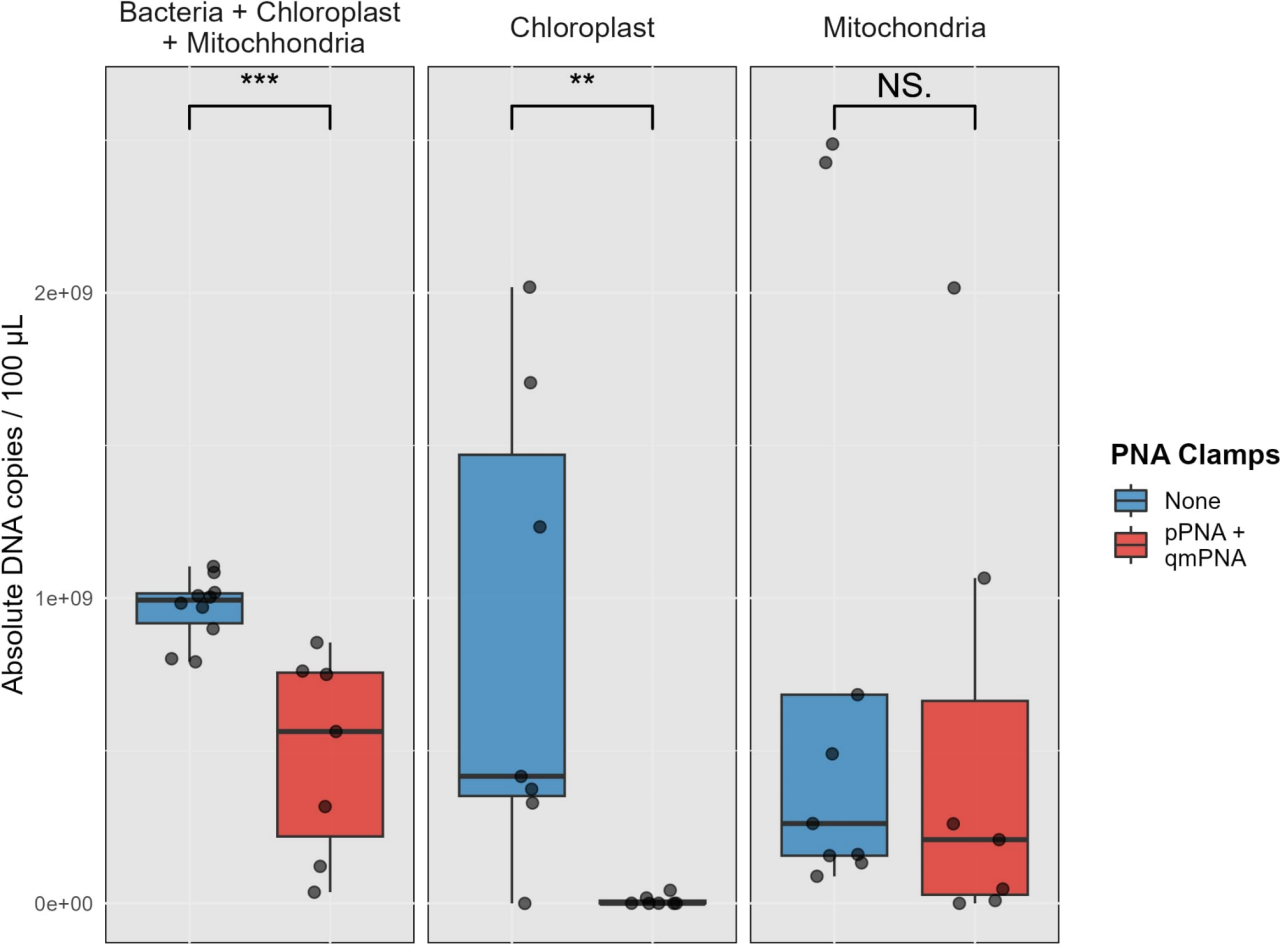
Quantification of absolute DNA copies of bacteria, host chloroplast and host mitochondria with and without PNA clamps during PCR via a qPCR assay. Quantification was performed on 16S rRNA gene PCR amplicons of *Q. petraea* and *Q. robur* leaf and bark samples, without and with PNA clamps added during PCR. A SYBR green and two single-plex probe-based TaqMan assays targeting host chloroplast and mitochondria DNA were conducted on the PCR amplicons. Stars indicate significant differences (p-value < 0.05, Wilcoxon rank sum test)

The effectiveness of PNA clamps in inhibiting PCR amplification of host chloroplast and mitochondria sequences was further quantitatively assessed and validated using probe-based qPCR TaqMan assays. The addition of PNA clamps during 16S rRNA gene PCR amplification led to a decrease in total PCR amplification, host chloroplast and mitochondria amplicons (Fig. 3). In leaf tissue, there was an average reduction of 23%, 97%, and 38% for all PCR amplicons, host chloroplast and host mitochondria respectively. In bark tissue, there was an average reduction of 67%, 98% and 17% in all PCR amplicons, host chloroplast and host mitochondria respectively. In root tissue, there was an average reduction of 40%, 100% and 88% in all PCR amplicons, host chloroplast and host mitochondria respectively.

The inclusion of PNA clamps resulted in a significant decrease in both overall PCR amplification (p-value < 0.01) and PCR amplification of host chloroplasts sequences (p-value = 0.01). However, there was no significant reduction observed in PCR amplification of host mitochondria sequences (p-value = 0.35).

## Discussion

Due to the co-amplification of host chloroplast and mitochondrial rRNA sequences during PCR, a lower abundance of plant-associated bacterial 16S rRNA gene reads are often obtained from PCR-based sequencing studies, leading to underrepresentation of the bacterial community (Lutz et al., 2011; Hanshew et al., 2013). In this study, we tested the universal pPNA clamp (Lundberg et al., 2013) and a newly designed qmPNA clamp for their ability to respectively minimise host chloroplast and mitochondria PCR co-amplification in oak 16S rRNA gene sequencing. Our results indicate that the addition of PNA clamps significantly reduced levels of host DNA co-amplification, leading to increased amplification of bacterial 16S rRNA gene sequences and improved assessment of bacterial community diversity and composition.

The commercially available pPNA clamp was highly effective in inhibiting host chloroplast amplification in PCR 16S rRNA gene sequencing achieving an average reduction of 82% in leaf and 74% in bark tissues. These results were further validated by the qPCR TaqMan assays, which showed an average 98% reduction in host chloroplast amplification across all tissues. However, this reduction of host chloroplast amplification resulted in a relative increase in host mitochondria when the pPNA clamp was used alone, leading to mitochondria ASVs making up on average 96% and 98% of all reads in leaf and bark tissue respectively.

Although there is a commercially available PNA clamp targeting plant mitochondria, its sequence did not match our mitochondrial ASVs from *Q. robur* and *Q. petraea* which led us to design a *Quercus* specific mitochondria clamp. Our custom-made clamp (qmPNA) was designed based on the most abundant host mitochondrial ASVs and this custom clamp could bind in silico to 18 out of 93 mitochondrial ASVs identified in this study. When the qmPNA clamp was used alongside the pPNA clamp during PCR 16S rRNA gene sequencing, the increase in host mitochondria amplification was limited to 9% and 40% in leaf and bark tissues, respectively. The fact that the host mitochondrial reads still showed a relative increase when adding both PNA clamps compared to no clamps is mostly due to a relative rise in reads assigned to host mitochondrial ASVs which the qmPNA clamp was not designed to target (58 out of the 93 mitochondria ASVs). This could potentially be resolved by designing a mixture of several PNA clamps which target a wider range of host mitochondria sequences.

Using the mitochondrial ASVs identified in this study, two additional variants of mPNA clamps have been identified: qmPNAv2 (“GTGGAATTTCGTGTGTA”) and qmPNAv3 (“GGTTGAAAGTGAAAGTC”). In silico analysis shows that qmPNAv2 and qmPNAv3 bind with 100% specificity to 27 and 33 unique mitochondrial ASVs, respectively. Combined with the mPNA clamp tested in this study, the three clamps collectively bind to 78 out of 93 host mitochondrial ASVs. This suggests a set of three mPNA clamps could theoretically be used to target most of the host oak mitochondrial ASVs, further reducing host mitochondrial contamination.

An effective PNA clamp is generally expected to have a melting temperature (Tm) exceeding 72 °C. The Tm of the pPNA and qmPNA clamps used in this study were 82 °C and 76 °C, respectively. The PNA clamp binding temperature is typically 5–10 °C below its Tm. Accordingly, a binding temperature of 68 °C was selected in this study to facilitate efficient qmPNA clamp binding while remaining above the primer Tm of 66 °C. Here, the binding and primer annealing steps were lower than those of other studies [27], which increases the risk of non-specific binding. However, the lack of change between the no PNA and both PNA clamp groups in the root samples suggests minimum non-specific binding. Future tests could experimentally validate this by designing DNA strands with 1–5 mismatches to designed PNA clamps and checking their amplification during PCR.

The qmPNA clamp was less effective at inhibiting the amplification of the most abundant host mitochondrial ASV compared to the pPNA clamp’s inhibition of the most abundant host chloroplast ASV. When the pPNA clamp was added, the most abundant host chloroplast ASV, which initially made up 99% of all host chloroplast sequencing reads, was completely inhibited during PCR. In contrast, the most abundant host mitochondrial ASV remained the most prevalent even after qmPNA clamp treatment, reducing from 97 to 62% of the total host mitochondrial reads. Although the qmPNA clamp was not as effective, this reduction does suggest that the qmPNA clamp provided some level of inhibition, and to improve its effectiveness, increasing the final concentration of the qmPNA in the PCR reaction mixture could be considered. PNAbio states that the most common concentrations used for PNA clamps are between 0.4 and 2 µM. However, a study showed concentrations of up to 3.75 µM were optimum for the rhizosphere [40]. Considering the high abundance of host chloroplast and mitochondria co-amplification observed in this study, PNA clamp concentrations of up to 4–5 µM could be tested. However, at such high concentrations, issues of non-specific binding may become more prevalent and the clamp specificity could be tested as described above.

Despite an increase in total host mitochondrial reads during sequencing, the addition of PNA clamps allowed PCR primers to more effectively amplify bacterial sequences. Consequently, there was an increase in bacterial 16S rRNA gene reads by 72%, 35%, and 17% in leaf, bark, and root samples, respectively. This improvement enhanced the resolution of the microbial community composition in leaf and bark samples from 21 to 8 to 126 and 226 ASVs, respectively.

On the other hand, the root samples, which only contain few host chloroplast and mitochondria, did not show significant change in alpha and beta diversity, and demonstrated nearly identical relative abundance profiles with and without PNA clamps. This suggests that the PNA clamps did not add any taxonomic bias to the observed bacterial community, and other studies support this [23]. Some alpha and beta diversity differences were observed between the no PNA and both PNA clamp groups in the leaf and bark tissue. The change in phyla relative abundance in leaf and bark is likely due to the very low number of bacterial reads without PNA clamps, sometimes as low as nine reads per sample. See supplementary Table 4 for more information. These low numbers of reads bias the bacterial community composition, whilst, with the clamps, there is a much higher sequencing depth, which allows for more reliable bacterial composition analysis. This is supported by the consistency of bacterial phyla relative abundance with and without clamps in the root samples, suggesting that the addition of the clamps does not bias the bacterial community composition.

The sequencing depth in this study was on average 162 443 filtered reads per sample, which is higher than typical plant-microbiota-associated 16S rRNA gene sequencing studies that range between 2000 and 40 000 reads per sample [20, 41, 42]. Even with high sequencing depth, leaf and bark tissues without PNA clamps exhibited far fewer unique ASVs, with 21 and 8 respectively. In contrast, the same samples with PNA clamps showed a significant increase in unique ASVs, with 126 in leaf tissue and 226 in bark tissue. This suggests increasing sequencing depth cannot resolve the issue of host DNA co-amplification. Additionally, previous studies have shown that DNA extraction yields also do not impact rates of host chloroplast and mitochondria reads in sequencing (Fitzpatrick et al., 2018), which is logical since the issue lies within the PCR step.

In this study, qPCR TaqMan assays were used to validate the PCR 16S rRNA gene sequencing results and as a faster method for testing and optimising PNA clamps towards inhibiting host chloroplast and mitochondria co-amplification. These assays, like the sequencing data, revealed a significant reduction in host chloroplast DNA copy numbers. However, while sequencing data showed an increase in host mitochondria amplification, the qPCR assays indicated a slight reduction. This discrepancy arises from the limitation of the qPCR probes being designed with the same sequence as the PNA clamps, and thus only binds to a certain proportion of host chloroplast and mitochondrial sequences. Mitochondrial ASVs which were not targeted by the PNA clamps and increased in abundance after addition of both PNA clamps in the PCR 16S rRNA gene sequencing results are thus not picked up by the probe-based qPCR assay, leading to an incomplete picture of the PCR amplification.

Our study demonstrated that a single ASV could constitute up to 99% of all the host chloroplast or mitochondria reads, and in these circumstances qPCR assays could be a valuable tool in optimising PNA clamps against specific host sequences. Given the diverse range of host chloroplast and mitochondria sequences across different plant species [30] and the reduced effectiveness of PNA clamps with even a single base pair mismatch [31], it is crucial to design host-specific PNA clamps and to thoroughly test effectiveness for each individual application. Through such testing, PNA clamps could be used as method to reduce host chloroplast and mitochondria co-amplification and increase the diversity captured by plant associated bacterial 16S rRNA gene sequencing, as shown in this study.

## Conclusion

Overall, this study shows that PNA clamps can effectively reduce host chloroplast and mitochondria PCR amplification and improve assessment of the detected bacterial diversity in *Quercus petraea* and *Quercus robur* bacterial 16S rRNA gene sequencing studies. Future research should focus on optimising the PNA clamp concentrations and possibly developing a variety of PNA clamps to target different sequence variants of host mitochondrial sequences.

## Electronic supplementary material

Below is the link to the electronic supplementary material.

Supplementary Material 1

## Data Availability

The raw sequence data were deposited in the European Nucleotide Archive (ENA) under the study accession number PRJEB78505; https://www.ebi.ac.uk/ena/browser/view/PRJEB78505. The code and processed data for sequencing and qPCR can be found at https://github.com/Usman-Hussain567/PNA-Clamp-Optimisation-for-Oak-Microbiome-Studies.
