# Supplementary material for: Peptide nucleic acid (PNA) clamps reduce amplification of host chloroplast and mitochondria rRNA gene sequences and increase detected diversity in 16S rRNA gene profiling analysis of oak-associated microbiota
Source: Environ Microbiome. 2025 Jan 28;20:14. doi: 10.1186/s40793-025-00674-w (PMC11773970; doi:10.1186/s40793-025-00674-w)
Supplement: Supplementary file 1 — Supplementary Material 1 [file 40793_2025_674_MOESM1_ESM.docx]

**Supplementary Material**

**Correcting for tag-jumping**

Upon inspection of the amplicon sequencing data, it became clear that tag jumping (Schnell, Bohman and Gilbert 2015) had occurred during library preparation, which due to the combinatorial dual indexing used in the study, allowed sequences to jump between samples, leading to false-positive detections. To correct the data for these false positives, we modified the approach from (Rodriguez-Martinez *et al.*, (2023). Assuming the data from unused tag jumping combinations represented an unbiased measurement of tag jumping rates, we fit a Bayesian negative binomial regression model for each sequencing library in the Bayesian modelling package “brms” (Bürkner 2017) to model the expected rate of tag jumping in these unused tag combinations for each ASV. We captured the geometric element of tag jumping by modelling the tag jumping rate as a function of `log(Reads in tag matrix column * Reads in tag matrix row)`. The model was hierarchical, with a varying intercept and slope for each ASV. We used weakly-informative priors on all parameters to constrain the model, with a normal(0, 3) prior on the fixed effects, an exponential(2) prior on the varying effect standard deviations, and an lkj(3) prior (Lewandowski, Kurowicka and Joe 2009) on the correlation between the varying intercept and slope. For each used tag combination and ASV, we then predicted the upper 95% CI for the number of reads expected if there were no reads present originally in that combination. If the observed number of reads was higher than this limit, we kept the data, and if it was lower, the reads for that ASV in that sample were set to 0. This allowed us to take a conservative approach, retaining only counts where positives were highly plausible.

| ASV | Sequence |
| --- | --- |
| Chloroplast | 5’ – TACAGAGGATGCAAGCGTTATCCGGAATGATTGGGCGTAAAGCGTCTGTAGGTGGCTTTTTAAGTCCGCCGTCAAATCCCAGGGCTCAACCCTGGACAGGCGGTGGAAACTACCAAGCTGGAGTACGGTAGGGGCAGAGGGAATTTCCGGTGGAGCGGTGAAATGCGTAGAGATCGGAAAGAACACCAACGGCGAAAGCACTCTGCTGGGCCGACACTGACACTGAGAGACGAAAGCTAGGGGAGCGAATGGG – 3’ |
| Mitochondria | 5’ – GACGGGGGGGGCAAGTGTTCTTCGGAATGACTGGGCGTAAAGGGCACGTAGGCGGTGAATCGGGTTGAAAGTGAAAGTCGCCAAAAACTGGTGGAATGCTCTCGAAACCAATTCACTTGAGTGAGACAGAGGAGAGTGGAATTTCGTGTGTAGGGGTGAAATCCGGAGATCTACGAAGGAACGCCAAAAGCGAAGGCAGCTCTCTGGGTCCCTACCGACGCTGGAGTGCGAAAGCATGGGGAGCGAACGGG – 3’ |

**Table S1. The most abundant chloroplast and mitochondria ASVs** This table gives the V4 16S rRNA gene sequences of the most abundant chloroplast and mitochondria ASVs obtained from leaf, bark and root samples. These were consistent from both *Quercus robur* and *Quercus petraea* in our preliminary data when PNA clamps were not added during PCR.

| Name | Sequence |
| --- | --- |
| 16S_F515DI1 | CCTAAACTACGG GTGBCAGCMGCCGCGGTAA |
| 16S_F515DI2 | TGCAGATCCAAC GTGBCAGCMGCCGCGGTAA |
| 16S_F515DI3 | CCATCACATAGG GTGBCAGCMGCCGCGGTAA |
| 16S_F515DI4 | GTGGTATGGGAG T GTGBCAGCMGCCGCGGTAA |
| 16S_F515DI5 | ACTTTAAGGGTG T GTGBCAGCMGCCGCGGTAA |
| 16S_F515DI6 | GAGCAACATCCT T GTGBCAGCMGCCGCGGTAA |
| 16S_F515DI7 | TGTTGCGTTTCT GT GTGBCAGCMGCCGCGGTAA |
| 16S_F515DI8 | ATGTCCGACCAA GT GTGBCAGCMGCCGCGGTAA |
| 16S_F515DI9 | AGGTACGCAATT GT GTGBCAGCMGCCGCGGTAA |
| 16S_F515DI10 | ACAGCCACCCAT CGA GTGBCAGCMGCCGCGGTAA |
| 16S_F515DI11 | TGTCTCGCAAGC CGA GTGBCAGCMGCCGCGGTAA |
| 16S_F515DI12 | GAGGAGTAAAGC CGA GTGBCAGCMGCCGCGGTAA |
| 16S_F515DI13 | GTTACGTGGTTG ATGA GTGBCAGCMGCCGCGGTAA |
| 16S_F515DI14 | TACCGCCTCGGA ATGA GTGBCAGCMGCCGCGGTAA |
| 16S_F515DI15 | CGTAAGATGCCT ATGA GTGBCAGCMGCCGCGGTAA |
| 16S_F515DI16 | TACCGGCTTGCA TGCGA GTGBCAGCMGCCGCGGTAA |
| 16S_F515DI17 | ATCTAGTGGCAA TGCGA GTGBCAGCMGCCGCGGTAA |
| 16S_F515DI18 | CCAGGGACTTCT TGCGT GTGBCAGCMGCCGCGGTAA |
| 16S_F515DI19 | CACCTTACCTTA GAGTGG GTGBCAGCMGCCGCGGTAA |
| 16S_F515DI20 | ATAGTTAGGGCT GAGTGG GTGBCAGCMGCCGCGGTAA |
| 16S_F515DI21 | GCACTTCATTTC GAGTGG GTGBCAGCMGCCGCGGTAA |
| 16S_F515DI22 | TTAACTGGAAGC CCTGTGG GTGBCAGCMGCCGCGGTAA |
| 16S_F515DI23 | CGCGGTTACTAA CCTGGAG GTGBCAGCMGCCGCGGTAA |
| 16S_F515DI24 | GAGACTATATGC CCTGGAG GTGBCAGCMGCCGCGGTAA |
| 16S_R806DI1 | CCTAAACTACGG GGACTACHVGGGTWTCTAAT |
| 16S_R806DI2 | TGCAGATCCAAC GGACTACHVGGGTWTCTAAT |
| 16S_R806DI3 | CCATCACATAGG GGACTACHVGGGTWTCTAAT |
| 16S_R806DI4 | GTGGTATGGGAG A GGACTACHVGGGTWTCTAAT |
| 16S_R806DI5 | ACTTTAAGGGTG A GGACTACHVGGGTWTCTAAT |
| 16S_R806DI6 | GAGCAACATCCT A GGACTACHVGGGTWTCTAAT |
| 16S_R806DI7 | TGTTGCGTTTCT TC GGACTACHVGGGTWTCTAAT |
| 16S_R806DI8 | ATGTCCGACCAA TC GGACTACHVGGGTWTCTAAT |
| 16S_R806DI9 | AGGTACGCAATT TC GGACTACHVGGGTWTCTAAT |
| 16S_R806DI10 | ACAGCCACCCAT CTA GGACTACHVGGGTWTCTAA |
| 16S_R806DI11 | TGTCTCGCAAGC CTA GGACTACHVGGGTWTCTAAT |
| 16S_R806DI12 | GAGGAGTAAAGC CTA GGACTACHVGGGTWTCTAAT |
| 16S_R806DI13 | GTTACGTGGTTG GATA GGACTACHVGGGTWTCTAAT |
| 16S_R806DI14 | TACCGCCTCGGA GATA GGACTACHVGGGTWTCTAAT |
| 16S_R806DI15 | CGTAAGATGCCT GATA GGACTACHVGGGTWTCTAAT |
| 16S_R806DI16 | TACCGGCTTGCA ACTCA GGACTACHVGGGTWTCTAAT |
| 16S_R806DI17 | ATCTAGTGGCAA ACTCA GGACTACHVGGGTWTCTAAT |
| 16S_R806DI18 | CCAGGGACTTCT ACTCA GGACTACHVGGGTWTCTAAT |
| 16S_R806DI19 | CACCTTACCTTA TTCTCT GGACTACHVGGGTWTCTAAT |
| 16S_R806DI20 | ATAGTTAGGGCT TTCTCT GGACTACHVGGGTWTCTAAT |
| 16S_R806DI21 | GCACTTCATTTC TTCTCT GGACTACHVGGGTWTCTAAT |
| 16S_R806DI22 | TTAACTGGAAGC CACTTCT GGACTACHVGGGTWTCTAAT |
| 16S_R806DI23 | CGCGGTTACTAA CACTTCT GGACTACHVGGGTWTCTAAT |
| 16S_R806DI24 | GAGACTATATGC CACTTCT GGACTACHVGGGTWTCTAAT |

**Table S2**. **Sequences of forward and reverse bacterial 16S rRNA gene primers** Table showing the sequences of the tags and spacers of the forward and reverse primers. The names of the forward primers start with 16S_F and the names of the reverse primers start with 16S_R.


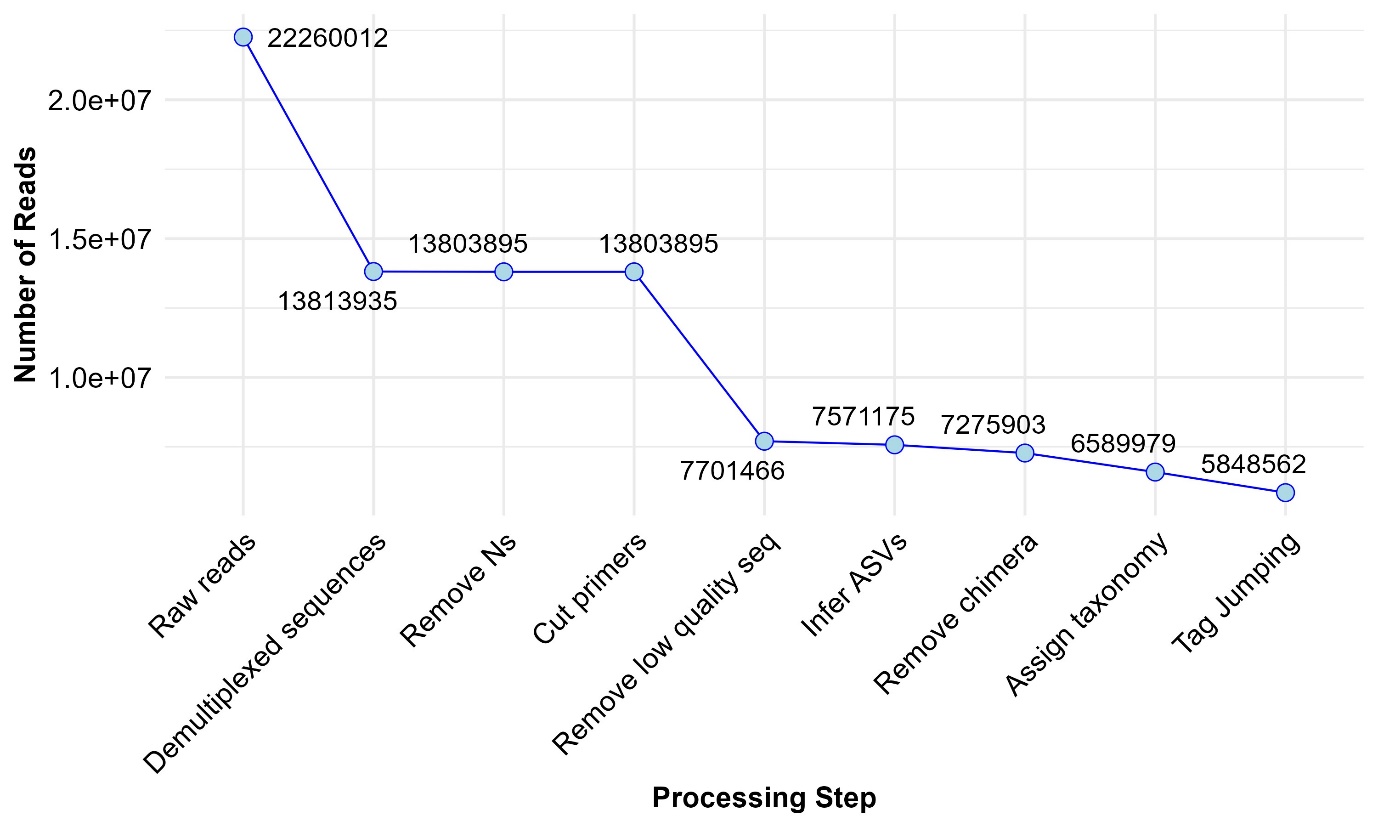


**Figure S1.** **Summary of read loss count through filtering.** A graph illustrating the number of sequencing reads remaining after each bioinformatic filtering step.

| ASV | Sequence |
| --- | --- |
| Bacteria | 5' - GTGGCAGCAGCCGCGGTAACCTGTTTGCTCCCCACGCTGTCGTGCCTCAGCGTCAGTTCCGGACCAGTGAGCCGCCTTCGCCACCGGTGTTCTTGCGAATATCTACGAATTTCACCTCTACACTCGCAGTTCCACTCACCTCTTCCGGACTCAAGATCCCCAGTATCAAAGGCAGTTCCGAGGTTGAGCCTCGGGATTTCACCCCTGACTTAAGAATCCGCCTACGCACCCTATTAGATACCCATGTAGTCC - 3' |
| Chloroplast | 5'- GTGGCAGCAGCCGCGGTAACCATTCGCTCCCCTAGCTTTCGTCTCTCAGTGTCAGTGTCGGCCCAGCAGAGTGCTTTCGCCGTTGGTGTTCTTTCCGATCTCTACGCATTTCACCGCTCCACCGGAAATTCCCTCTGCCCCTACCGTACTCCAGCTTGGTAGTTTCCACCGCCTGTCCAGGGTTGAGCCCTGGGATTTGACGGCGGACTTAAAAAGCCACCTACAGACGCTTTACGCCCAATCATTCCGGATAACGCTTGCATCCTCTGTAATTAGATACCCATGTAGTCC - 3' |
| Mitochondria | 5' - GTGGCAGCAGCCGCGGTAACCCGTTCGCTCCCCATGCTTTCGCACTCCAGCGTCGGTAGGGACCCAGAGAGCTGCCTTCGCTTTTGGCGTTCCTTCGTAGATCTCCGGATTTCACCCCTACACACGAAATTCCACTCTCCTCTGTCTCACTCAAGTGAATTGGTTTCGAGAGCATTCCACCAGTTTTTGGCGACTTTCACTTTCAACCCGATTCACCGCCTACGTGCCCTTTACGCCCAGTCATTCCGAAGAACACTTGCCCCCCCCGTCATTAGATACCCATGTAGTCC - 3' |

**Table S3. The sequences of the gBlocks used for qPCR standards.** The gBlock sequences used for the qPCR standards were selected from the most abundant chloroplast, mitochondria, and true bacterial ASVs. These sequences were obtained from preliminary data of leaf, bark, and root samples of *Quercus robur* and *Quercus petraea* when PNA clamps were not added during PCR


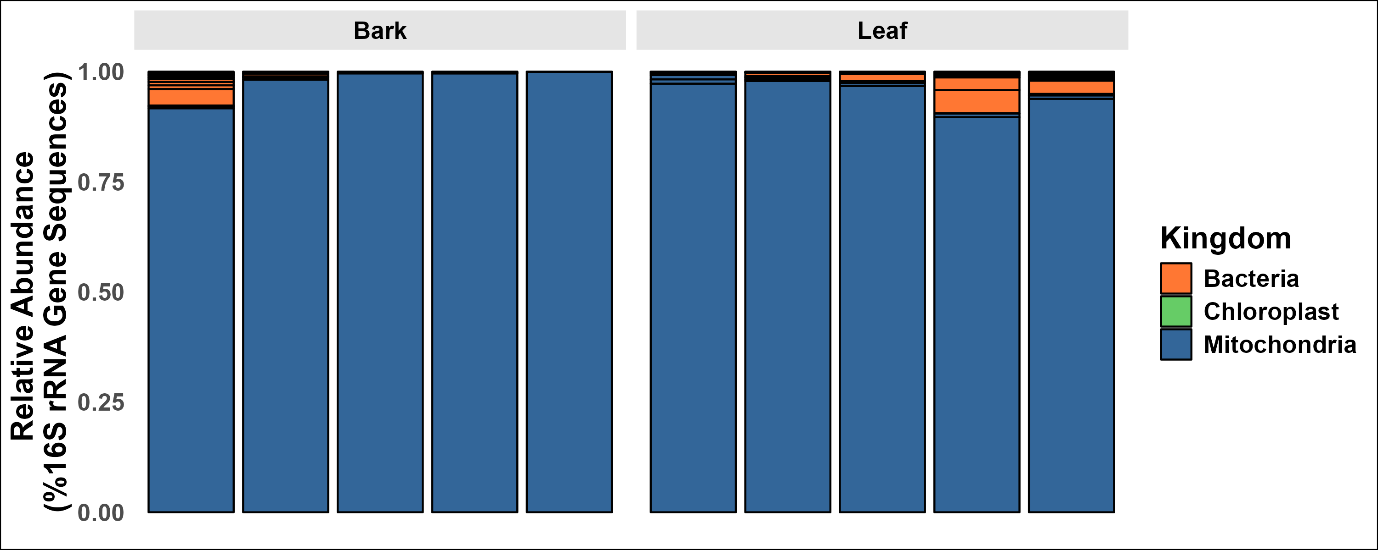


**Figure S2.** **Relative abundance of bacteria, host chloroplast and host mitochondria sequencing reads in oak tissue samples with just the pPNA clamps added during PCR.** To observe the impact of not using the mPNA clamp, PCR was conducted on a subset of leaf and bark samples without the mPNA clamp and just the pPNA clamp. Observing the percentage relative abundances of 16S rRNA gene sequences from oak leaf and bark samples with just the pPNA clamp included in PCR. The relative abundances of amplicon sequence variants (ASVs) identified as host plastid chloroplast (green) and mitochondria (blue) are presented alongside bacterial sequences (orange). On average, the leaf tissue constituted 0.19 % (±0.19 %), 96 % (±3.6 %) and 3.6 % (± 3.8 %) of chloroplast, mitochondria and bacterial 16S rRNA gene sequences respectively. Bark samples were made up of on average 0.02 % (± 0.04 %), 98 % (±3.4 %) and 1.8 % (±3.4 %) for chloroplast, mitochondria and bacterial 16S rRNA gene sequences respectively.

| Sample | Raw Reads | Chloro + Mito Removed (Bacterial Reads) |  |
| --- | --- | --- | --- |
| BS-B1_B | 115663 | 31943 | |
| BS-B1_N | 147072 | 9 | |
| BS-B2_B | 159361 | 28933 | |
| BS-B2_N | 243343 | 9 | |
| BS-B3_B | 332606 | 227147 | |
| BS-B3_N | 142498 | 105 | |
| BS-L1_B | 317021 | 303956 | |
| BS-L1_N | 125016 | 12718 | |
| BS-L2_B | 345297 | 288301 | |
| BS-L2_N | 322327 | 139 | |
| BS-L3_B | 143709 | 104032 | |
| BS-L3_N | 194173 | 748 | |
| BS-R1_B | 94360 | 93939 | |
| BS-R1_N | 45930 | 45928 | |
| BS-R2_B | 32193 | 31819 | |
| BS-R2_N | 147016 | 28693 | |
| BS-R3_B | 94892 | 94432 | |
| BS-R3_N | 109877 | 108496 | |
| FO-B1_B | 4500 | 716 | |
| FO-B1_N | 143617 | 28 | |
| FO-B2_B | 36290 | 14223 | |
| FO-B2_N | 263979 | 74 | |
| FO-B3_B | 77520 | 30448 | |
| FO-B3_N | 221885 | 57 | |
| FO-L1_B | 8533 | 6425 | |
| FO-L1_N | 191529 | 102 | |
| FO-L2_B | 157985 | 70097 | |
| FO-L2_N | 187935 | 89 | |
| FO-L3_B | 120153 | 87819 | |
| FO-L3_N | 168839 | 424 | |
| FO-R1_B | 195653 | 195653 | |
| FO-R1_N | 226841 | 226536 | |
| FO-R2_B | 118002 | 117884 | |
| FO-R2_N | 38157 | 29883 | |
| FO-R3_B | 308033 | 308030 | |
| FO-R3_N | 266152 | 266060 | |

Table S4. Showing the number of DNA sequences obtained for each sample before and after rarefying and removing host chloroplast and mitochondria contamination. BS = *Quercus petraea*, FO = *Quercus robur*; B(1/2/3) = Bark, L(1/2/3) = Leaf, R(1/2/3) = Root; _N = No PNA Clamp, _B = Both PNA Clamps.


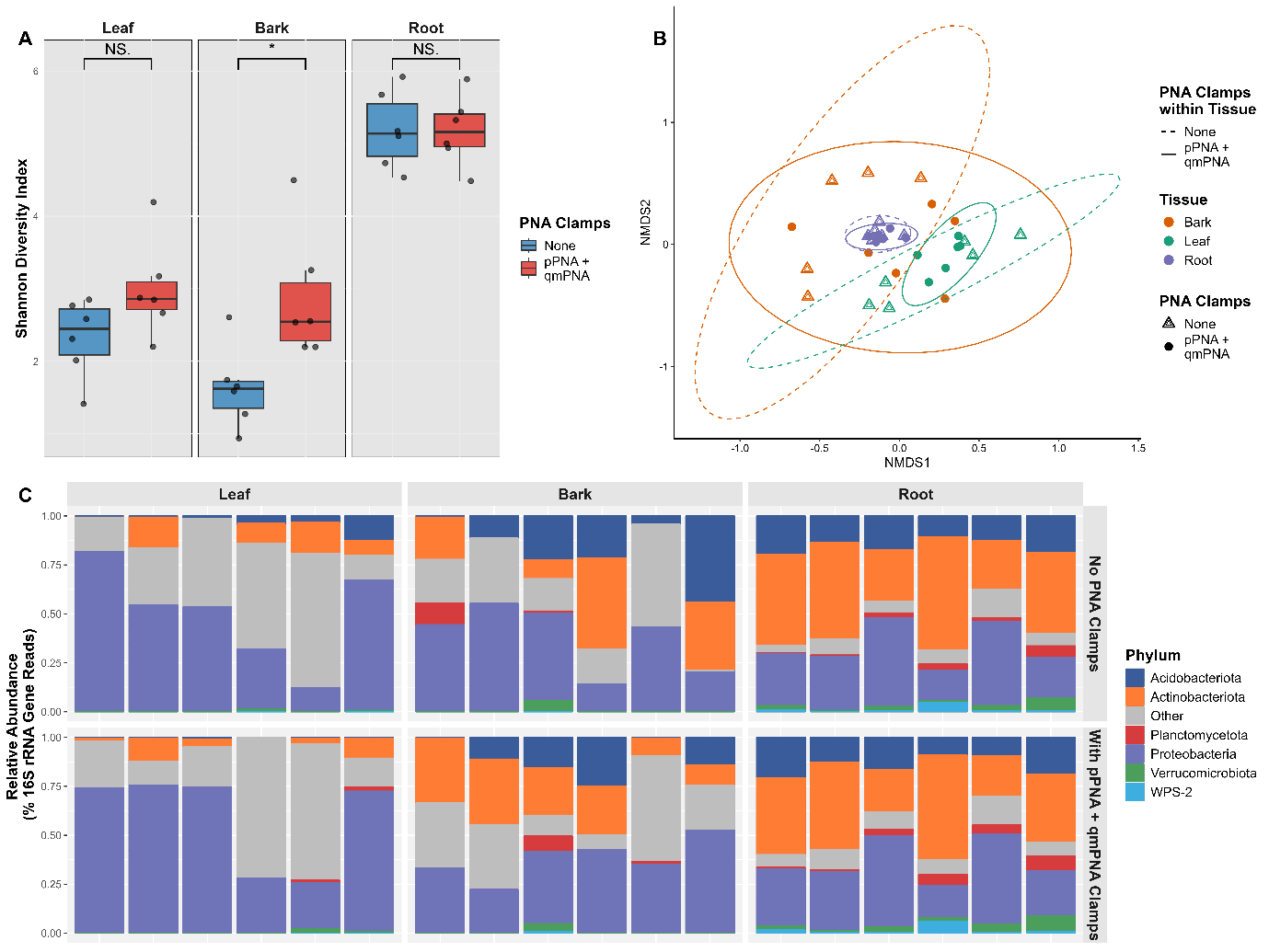


**Figure S3. Bacterial diversity and community profiles in oak tissue samples following 16S rRNA gene amplification with and without PNA clamps.** All reads assigned to the host chloroplast and mitochondria were removed before analysis. For each sample, the reads with and without clamps were rarefied to the lowest number to allow for comparison. **A.** Shannon diversity index with stars indicate significant differences **B.** Bray-Curtis Non-metric multidimensional scaling (NMDS). Solid (no PNA clamps) and dashed (pPNA + qmPNA clamps) ellipses represent 95% confidence intervals of normal multivariate distributions. **C.** Phyla relative abundance based on 16S rRNA gene reads. The top 6 most abundant phyla are presented, and the remaining phyla are categorised into Other (grey).

**References**

Bürkner, Paul Christian, ‘Brms: An R Package for Bayesian Multilevel Models Using Stan’, *Journal of Statistical Software*, 80/1 (2017)

Lewandowski, Daniel, Kurowicka, Dorota, and Joe, Harry, ‘Generating Random Correlation Matrices Based on Vines and Extended Onion Method’, *Journal of Multivariate Analysis*, 100/9 (2009), 1989–2001 <http://dx.doi.org/10.1016/j.jmva.2009.04.008>

Rodriguez-Martinez, Saul, Klaminder, Jonatan, Morlock, Marina A., Dalén, Love, and Huang, Doreen Yu Tuan, ‘The Topological Nature of Tag Jumping in Environmental DNA Metabarcoding Studies’, *Molecular Ecology Resources*, 23/3 (2023), 621–31

Schnell, Ida Bærholm, Bohman, Kristine, and Gilbert, M. Thomas P., ‘Tag Jumps Illuminated – Reducing Sequence-to-Sample Misidentifications in Metabarcoding Studies’, *Molecular Ecology Resources*, 2015
